# Detachment mechanism and reduced evaporation of an evaporative NaCl salt crust

**DOI:** 10.1038/s41598-022-11541-w

**Published:** 2022-05-06

**Authors:** G. Licsandru, C. Noiriel, S. Geoffroy, A. Abou-Chakra, P. Duru, M. Prat

**Affiliations:** 1 Institut de Mécanique des Fluides de Toulouse (IMFT), Université de Toulouse, CNRS-Toulouse, Toulouse, France; 2 GET (Géosciences Environnement Toulouse, Observatoire Midi Pyrénées, Université Paul Sabatier, CNRS, IRD, CNES, Université de Toulouse, Toulouse, France; 3 LMDC (Laboratoire Matériaux et Durabilité des Constructions), Université de Toulouse, INSAT, UPS, Toulouse, France

**Keywords:** Fluid dynamics, Civil engineering, Hydrology

## Abstract

Salt crusts forming at the surface of a porous medium are commonly observed in nature as well as on building materials and pieces of our cultural heritage where they represent a risk for the supporting substrate integrity. Previous research indicates that the salt crust can detach from the porous substrate and severely reduces the evaporation. However, the current understanding of the detachment mechanisms and the reduced evaporation is very limited. In the present experiment, we evidence dissolution–precipitation processes as key mechanisms in the detachment process. We also show that the crust remains wet and the observed reduced evaporation is explained by the formation of tiny pores in the nanometer range and the Kelvin effect. The resulting crust permeability is very low. Combined with previous results, this shows that the crust permeability is highly dependent on the crust formation conditions. More generally, salt structures in a water vapor concentration gradient are shown to be self-propelled systems capable to carry small objects such as, for instance, soil particles. Our study has significance for understanding the impact of salt crusts on evaporation and the associated important phenomena, such as soil salinization and porous material degradation inherent to salt crystallization.

## Introduction

Salt precipitation in porous media has gained attention in relation with several processes, such as evaporation in the critical zone^1–4^, soil and rock weathering^5,6^, arable soil salinization^7,8^ or monument and construction material degradation^9–11^. A key issue in this context is the impact of salt precipitation on evaporation^2,3,12–17^, especially when precipitation results in the formation of a salt crust at the porous medium surface as commonly observed in soils^18^ or building materials^19^. Salt crust formation can severely reduce evaporation^1–3,13,20^. However, the mechanisms leading to the evaporation reduction are yet to be unraveled. Also, the salt crust can disconnect from the soil surface with the formation of an air gap between the crust bottom and the porous medium^18,21^. However, the mechanisms leading to the air gap formation are not understood. Although it is tempting to relate the two observations and speculate that the reduced evaporation is due to the air gap formation, we show that this is not the case in our experiment.

## Experiment

**Figure 1:**
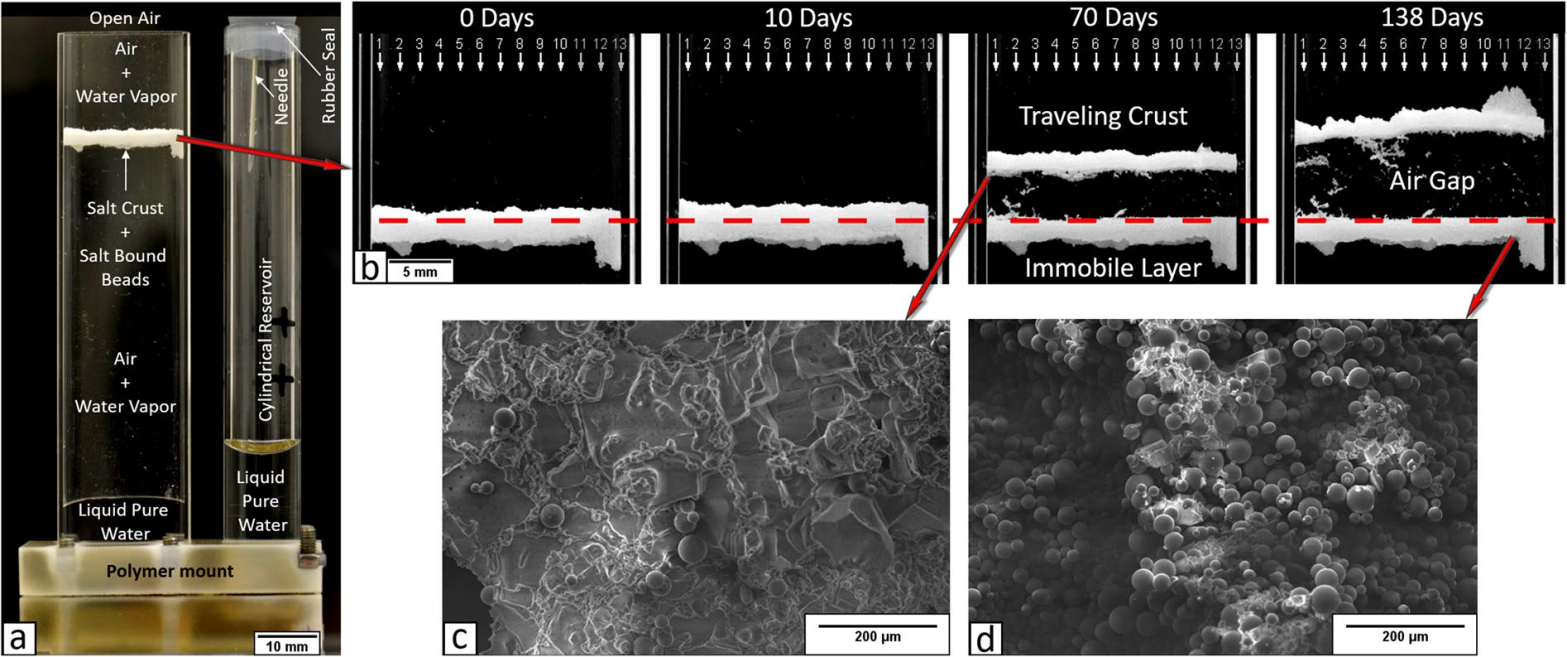
(**a**) Experimental setup at the beginning of the experiment, with the Hele–Shaw cell connected to the adjacent cylinder. (**b**) Upward displacement of the porous crust and detachment from the glass bead layer leading to the air gap formation. The red dashed line corresponds to the uppermost position of the glass bead packing partially filling the cell during the drying step in which the crust forms. (**c**,**d**) Scanning Electron Microscopy (SEM) observations of crust after the end of the experiment (**c**) detached porous salt crust and (**d**) lower immobile layer after crust detachment**.**

We present a dedicated 138 days experiment that focuses on the direct observation of the formation of the air gap between a salt crust and the porous medium surface, as well as the evaluation of mass loss due to evaporation. As shown in Fig. 1, the studied system consists in a salt crust suspended in a Hele–Shaw cell of 10 cm in height, and with a cross section of 2 cm × 0.2 cm. Initially, the cell was rendered hydrophobic by silanization prior to experiment to avoid as much as possible salt creeping^22,23^. The salt crust was formed prior to the experiment as a result of a 130-days drying in the glass Hele–Shaw cell packed with glass beads of diameter in the range of (1–50 μm). The packing was saturated with a 25% (by weight) NaCl aqueous solution, thus close to saturation (26.4% by weight). At the end of the drying process, the crust was formed at the packing top surface. Then, the non-cohesive glass beads underneath the crust region were carefully removed from the cell and the porous salt crust together with the upper surface bead packing was left suspended (Fig. 1a) in the cell.

The experiment starts with the supply of pure water in the adjacent glass cylinder reservoir (10 cm high and 1 cm in diameter) connected to the Hele–Shaw cell through a micro-channel in the polymeric base (Fig. 1a). The water level was modified twice in the cylinder. First, the level in the cylinder was set low to *h*_*llcyl.*_ ≈ 20 mm (measured from bottom) to avoid gas pressurization in the cell that could damage the crust. After 8 days, the water level was gradually raised to *h*_*llcyl.*_ ≈ 43 mm in 10 h. Then the experiment was continued for 130 days without any further water addition in the cylinder. Note that the cylinder is almost sealed at its top end to prevent for evaporation, while maintaining atmospheric pressure through the long needle visible in Fig. 1a. The experiment was performed in controlled environmental conditions, at room temperature *T* ≈ 22 °C and relative humidity around the cell ${H}_{r,inf}$ ≈ 39% (using Rotronic Higropalm 2 fitted with a Rotronic Hygroclip SP05 probe). The setup is set on a precision scale (Mettler Toledo AX205 with readability up to 10^–5^ g) in order to record the water mass loss due to evaporation every 100 s. Pictures of the experiment are taken at 1000 s intervals in portrait mode with a Nikon D800E camera at a resolution of 7360 × 4912 pixels. A total of about 12,000 pictures were taken, resulting in a data set of ~ 1.2 TB. The data set is used to follow the salt crust displacement dynamics, including the crust top and bottom position changes with time, as well as the liquid levels in the cell and the cylinder.

## Results

### Air gap formation

**Figure 2:**
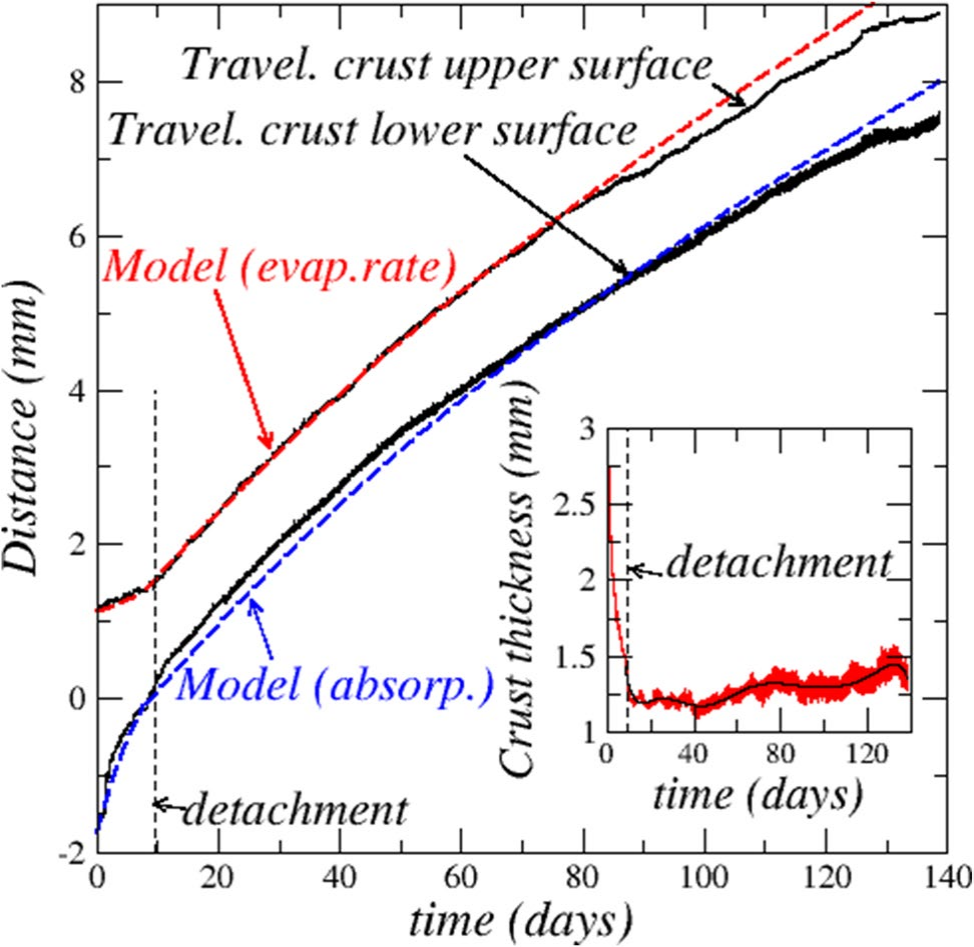
Travelling crust top and bottom surface mean positions as a function of time. The inset shows the changes in crust thickness as a function of time. The vertical dashed lines correspond to the complete detachment of the salt crust.

As illustrated in Fig. 1 (and Supplementary movie), the major event occurring in the experiment is the salt crust upward displacement and its full detachment from the porous medium. SEM observation in Fig. 1c shows that the moving layer is essentially formed by halite, i.e. NaCl crystals, with only a few entrapped glass beads. By contrast, as shown in Fig. 1d, the immobile layer is essentially composed of glass beads. This layer corresponds to the upper surface of the porous medium present in the Hele–Shaw cell during the drying step leading to the formation of the salt crust. Nevertheless, NaCl crystals are visible in Fig. 1d. They contribute to the immobile layer consolidation. As indicated in Fig. 2, detachment occurs after 9.5 days whereas the total crust upper surface displacement over the experiment duration is about 8 mm (distances in Fig. 2 are measured from the position of the top surface of glass bead packing which was present in the cell during the salt crust formation drying step. This position corresponds to the red dashed line in Fig. 1b). This is about 8 times the detached crust average thickness (Fig. 2, where the red plot in the inset corresponds to the raw data obtained by image processing whereas the black line is obtained by regression from the raw data). The resulting air gap width of about 8 mm is significant compared to the crust thickness and comparable to field observations^18^.

### Absorption and evaporation kinetics

**Figure 3:**
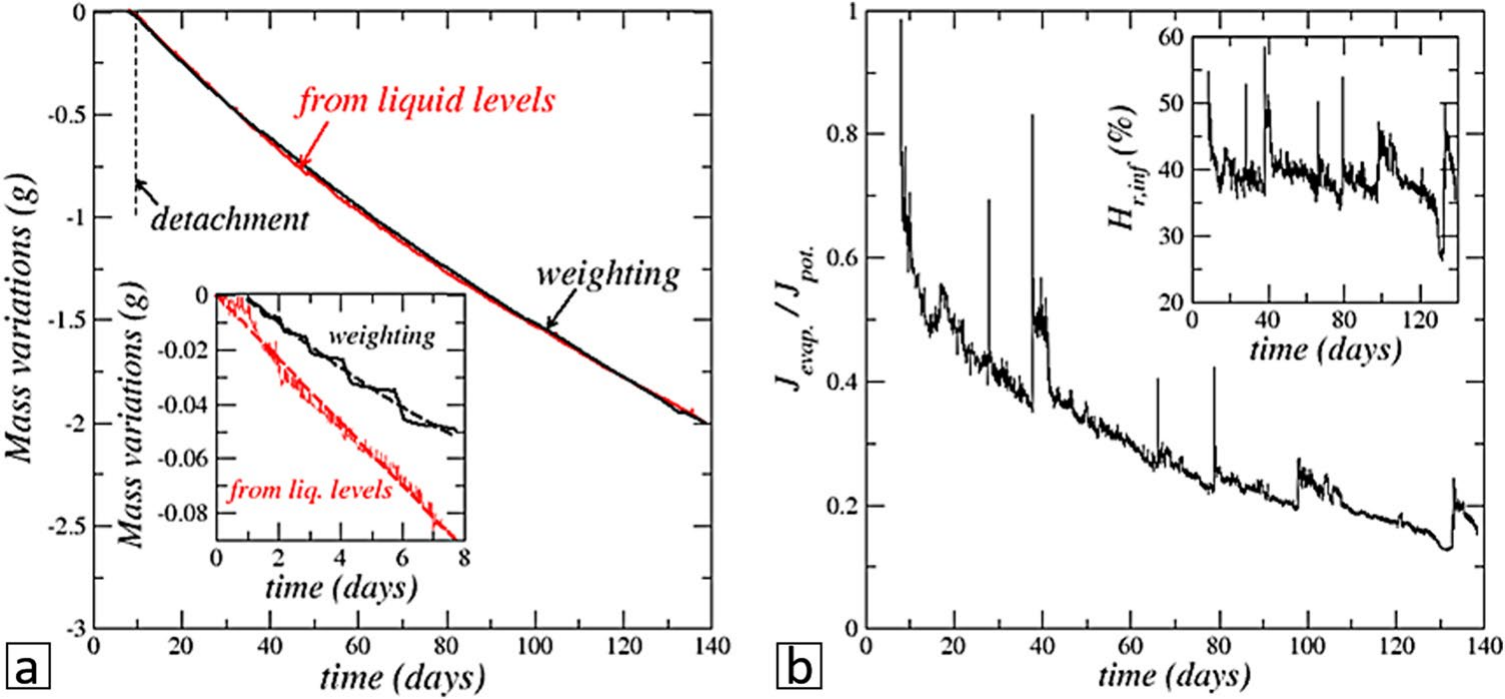
(**a**) Mass variations as a function of time after crust detachment. Comparison between the mass variation measured by weighting (dark solid line) and the one computed from the liquid level variations (red solid line) in the cell and adjacent cylinder. The inset shows the mass variations in the period before detachment (dashed lines are fits showing the main trends). (**b**) Comparison between the measured evaporation rate in the cell and the potential evaporation rate. The inset shows the relative humidity variation at the cell top.

The balance recorded mass variation (see “Methods”) is shown in Fig. 3 with the mass variation corresponding to the liquid level variations in the cell and adjacent cylinder. The liquid levels variation is considered as corresponding to the water absorbed by the crust since the level variations (outside of the two short filling periods) result from the mass transfer between the liquid water surface in the cell and the crust bottom surface. The set-up weight variation corresponds to the evaporation from the crust top surface since this is the net mass loss from the cell—cylinder assembly, recalling that the evaporation from the cylinder is negligible. Thus the evaporation flux from the crust top surface can be estimated as ${j}_{evap.}=-\frac{1}{A}\frac{d{m}_{weighting}}{dt}$ , whereas the absorption flux at the crust bottom surface is estimated as ${j}_{absorp.}=-\frac{1}{A}\frac{d{m}_{level}}{dt}=-\frac{\rho }{A}\frac{d}{dt}\left({Ah}_{llcell}+{A}_{cyl}{h}_{llcyl.}\right)$ , where ${h}_{llcell}$ is the liquid level in the cell and ${h}_{llcyl.}$ is the liquid level in the adjacent cylinder (visible in Fig. 1a), ${A}_{cyl}$ is the cylinder cross-section surface area,$A$ is the Hele–Shaw cell cross-section surface area and $\rho$ is the liquid water density.

As can be seen from Fig. 3a, ${m}_{weighting}\approx$ ${m}_{level}$ over the long crust upward displacement period after the detachment and thus ${j}_{evap.}\approx {j}_{absorp.}$ . By contrast, as shown in the inset in Fig. 3a, the mass variation computed from the liquid level variation in the adjacent cylinder and the cell before detachment is greater than the mass loss measured by weighting. Consequently, the water absorption rate by the crust is greater than the evaporation rate (${j}_{absorp.}>{j}_{evap.}$ ), and that specific period is referred to as the net absorption period.

### Evaporation is lower than the potential evaporation

The potential evaporation is the evaporation rate assuming that the crust top surface is covered by a saturated brine film. The relative humidity at the saturated brine surface is ${H}_{r,sat}$ = 0.75. From Fick’s law and under the usual quasi-steady assumption, the potential evaporation flux is thus given by ${j}_{evap.-pot.}={D}_{v}\frac{{M}_{v}}{RT}{p}_{vsat}\frac{\left({H}_{r,sat}-{H}_{r,inf}\right)}{{h}_{t}}$ , where *D*_*v*_ is the binary diffusion coefficient of the vapor in the gas phase, *R* is the universal gas constant, *M*_*v*_ is the vapor molecular weight, ${h}_{t}$ is the distance between the crust top surface and the cell top, ${H}_{r,inf}$ is the relative humidity measured at the cell top. The potential evaporation is compared to the evaporation rate determined from the measured set-up weight evolution during the long period after detachment in Fig. 3b. As can be seen, the evaporation rate is significantly lower than the potential evaporation, i.e. ${{j}_{evap.}< j}_{evap.-pot.}$ . The ratio ${{j}_{evap.}/ j}_{evap.-pot.}$ decreases all along the considered period and can be as low as about 15%.

## Analyses and explanations

### Crust upward displacement and gas pressure in the cell

**Figure 4:**
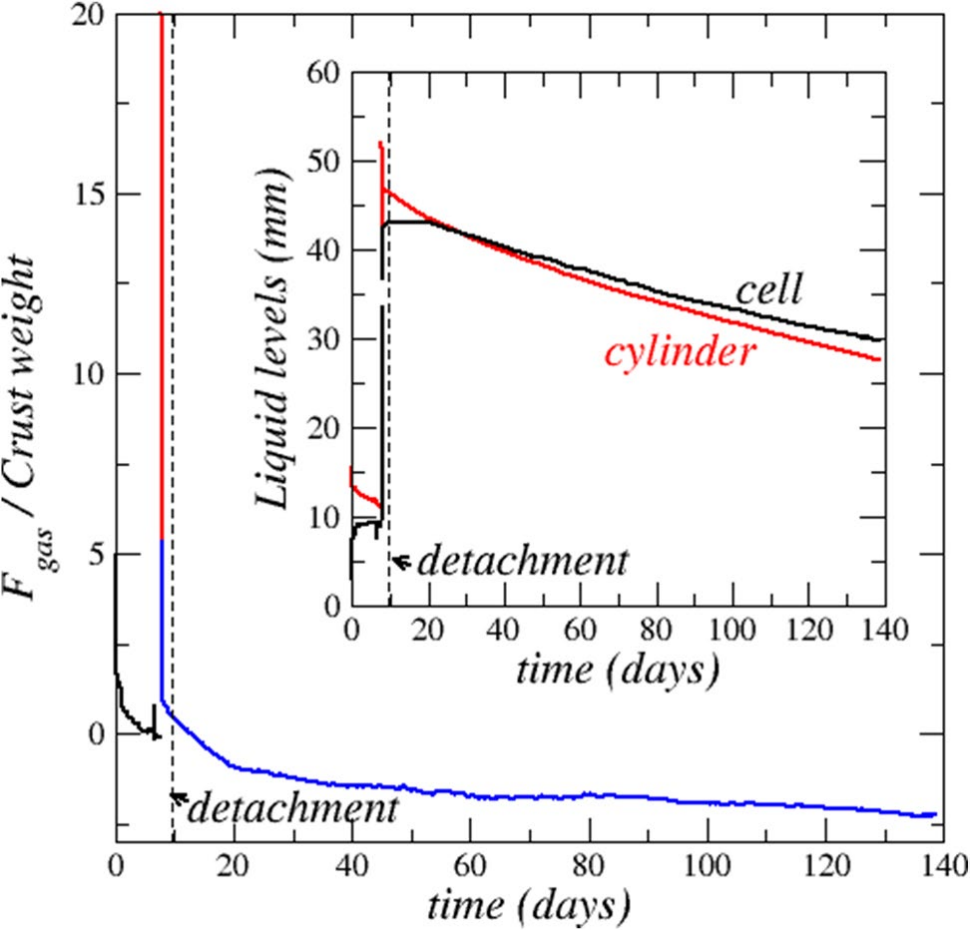
Evolution of the force exerted on the crust due to the gas pressure difference between the crust top and bottom. The force is scaled by the crust weight. The inset shows the variation of water levels in the cell (black) and the adjacent cylinder (red). Levels are measured from the bottom (top of polymeric base in Fig. 1a).

As can be seen from Fig. 1a, the liquid water level in the adjacent cylinder is higher than in the cell at the beginning of the experiment. Figure 4 shows that the cell and the adjacent cylinder liquid level dynamics is more complex than one could assume based on the system simplicity. Noting that the cell is rendered hydrophobic by silanization, the gas pressure in the cell can be determined assuming hydrostatic equilibrium between the liquid water in the cylinder and the cell: ${P}_{g-cell}={P}_{atm}+{(h}_{llcyl}-{h}_{llcell}){\rho }_{lw}g-\gamma cos\theta /a$ , where $\gamma$ is the surface tension (72 $\times$ 10^–3^ N/m),$\theta$ is the contact angle in the cell after silanization, i.e. $\theta \sim$ 110–120°, and *a* ≈ 1 mm is the cell aperture half. Based on the liquid level variations depicted in Fig. 4, the pressure in the cell can therefore be greater or lower than the atmospheric pressure. This indicates that the gas pressure below the crust can be different from the pressure above the crust. One can therefore wonder whether this pressure difference plays a role in the crust upward displacement. To clarify this point, the force exerted by the gas on the crust is computed as ${{F}_{g}={(P}_{g-cell}-{P}_{atm})A=((h}_{llcyl}-{h}_{llcell}){\rho }_{lw}g-\gamma cos\theta /a)A$ and is compared to the crust weight computed as ${W}_{g}=A{h}_{c}({\varepsilon }_{c}{\rho }_{s}+\left(1-{\varepsilon }_{c}\right){\rho }_{cr})g$ , where ${h}_{c}$ is the crust thickness, ${\varepsilon }_{c}$ is the crust porosity (taken equal to 0.25 as indicated by a mercury intrusion porosimetry measurement (see “Methods”)), ${\rho }_{s}$ is the NaCl saturated brine density (1200 kg/m^3^), ${\rho }_{cr}$ is the crystal density (2160 kg/m^3^), *g* is the acceleration due to gravity. The comparison in Fig. 4 shows that ${F}_{g}$ is actually negative (the gas pressure above the liquid water level in the cell is less than the atmospheric pressure) over most of the experiment. The conclusion is therefore that a gas pressurization effect in the cell cannot be invoked to explain the crust displacement. Furthermore, since the gas pressure tends eventually to be less than the atmospheric pressure (the computed difference is on the order of a few tens of Pa) as the gas volume between the liquid water at the cell bottom and the crust expands, it can be concluded that the moving crust gas permeability is extremely small.

### Air gap formation as a dissolution–precipitation process

**Figure 5:**
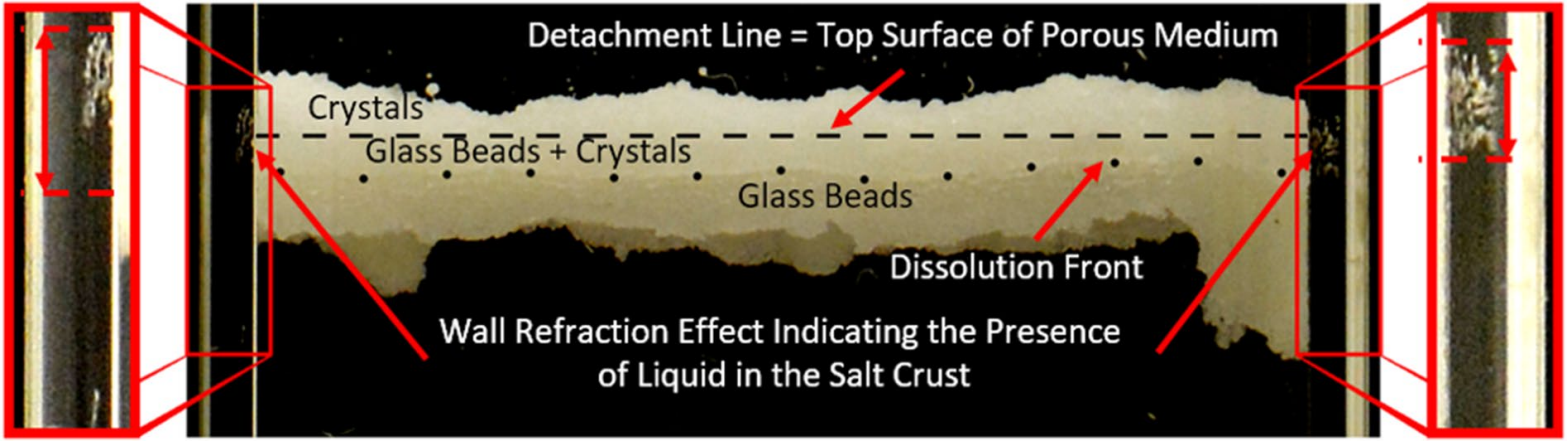
Observation of a greyish optical signal on the cell walls. The signal is visible on both sides when the crust moves upward, and indicates the presence of liquid water in the moving crust.

The remarkable upward displacement depicted in Fig. 1 is explained by a dissolution–precipitation process which implies that the crust is porous and wet. The porous nature of the crust is confirmed by mercury intrusion porosimetry (see below). Then, the question is whether the crust porosity is dry or filled with liquid. As mentioned earlier, the slight cell gas depressurization and the associated extremely low crust gas permeability is a first element in favor of a liquid saturated crust. The presence of liquid in the crust is also explicitly indicated by a wall reflection effect^24^ as illustrated in Fig. 5 (also visible in Supplementary movie). Hence, in the net absorption period, the brine gradually fills the pores in the crust until saturation.

The dissolution–precipitation process is due to vapor partial pressure gradients in the cell and associated diffusive transport. At the cell bottom, the vapor partial pressure above the pure liquid water interface is equal to the saturated vapor pressure, denoted by ${p}_{vsat}$ . At the cell top, the vapor partial pressure, ${p}_{v-cell-top}$ , is lower and very close to the vapor partial pressure imposed in the enclosure of controlled relative humidity and temperature (see “Methods”). Thus, ${{p}_{v-cell-top}={H}_{r,inf}p}_{vsat}$ where ${H}_{r,inf}$ is the enclosure relative humidity. In the solution saturated crust pores, the brine NaCl concentration is expected to be very close to thermodynamic equilibrium. The vapor partial pressure at the surface of a NaCl saturated aqueous solution is ${p}_{v-crust}=0.75 {p}_{vsat}$ . As a result, the situation in the cell is ${p}_{v-cell-bottom}>{{p}_{v-crust-surfaces}>p}_{v-cell-top}$ . These differences in vapor partial pressures induce a water transport by diffusion between (1) liquid water at the cell bottom and the salt crust bottom and (2) the salt crust upper surface and the cell top. Consequently, the crust absorbs water at its bottom as the result of water vapor condensation whereas evaporation takes place at its top. Since the crust thickness does not change appreciably after detachment (Fig. 2), water does not accumulate significantly within the crust during the crust displacement. As a result, the water absorbed at the crust bottom is transported within the crust pore space up to the crust top surface, where it evaporates. This is an indication that the crust pore network is percolating. Hence, solute transport takes place through the crust while water vapor condensation at the crust bottom leads to crystal deliquescence^25^, i.e. dissolution, and evaporation at its top induces precipitation.

**Figure 6:**
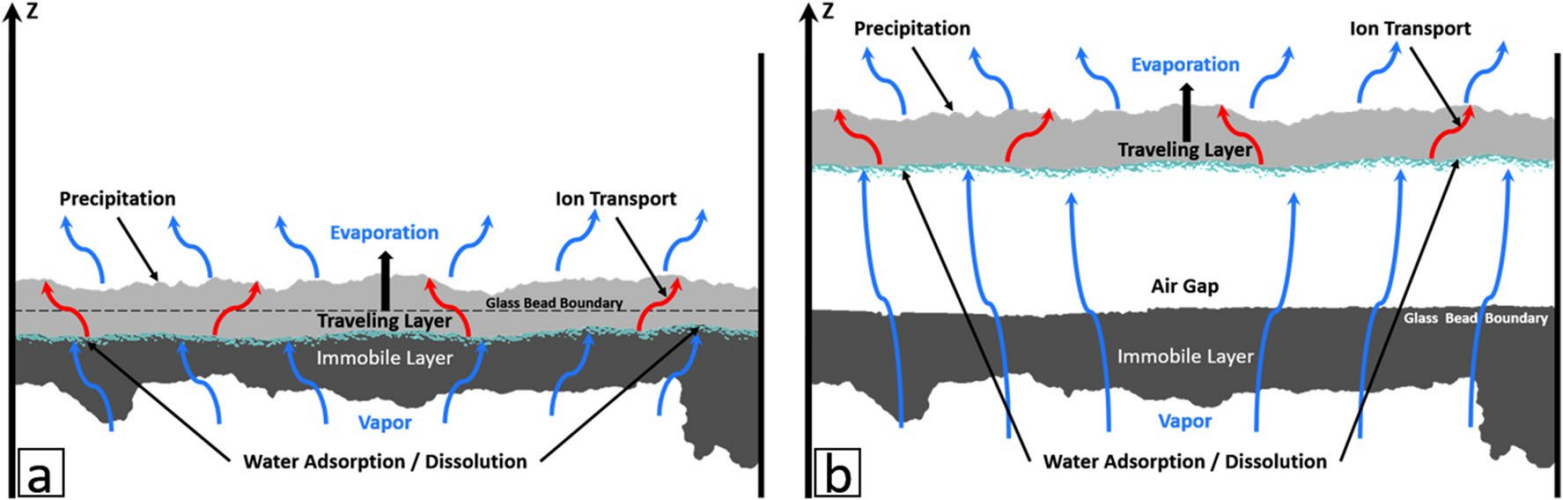
Schematic of displacement mechanism when the travelling layer is still embedded in the immobile layer (**a**) and after detachment (**b**).

This dissolution–precipitation displacement mechanism is sketched in Fig. 6. The displacement is quite slow. Thus, temperature variations associated with the liquid–vapor phase change processes occurring in the cell can be neglected. This scenario leads to the following relationships (adapted from ref.^26^) relating the displacement of the travelling crust top and bottom surfaces with the evaporation flux (top surface) or the absorption flux (bottom surface), $\frac{d{z}_{tct}}{dt}=\boldsymbol{ }\frac{{C}_{sat}{j}_{t}}{{\rho }_{cr}\left(1-{\varepsilon }_{c}\right)\left(1-{C}_{sat}\right)}\left[\frac{Da}{1+Da}\right]$ **,** $\frac{d{z}_{tcb}}{dt}=\boldsymbol{ }\frac{{C}_{sat}{j}_{b}}{{\rho }_{cr}\varepsilon \left(1-{\varepsilon }_{c}\right)\left(1-{C}_{sat}\right)}\left[\frac{Da}{1+Da}\right]$ where ${C}_{sat}$ is the solubility ion mass fraction (26.4%),${z}_{tct}$ is the travelling crust top surface mean position, ${z}_{tcb}$ is the travelling crust bottom surface mean position, ${j}_{t}$ is the evaporation flux at the travelling crust top surface,${j}_{b}$ is the absorption flux at the travelling crust bottom surface, ${\varepsilon }_{c}$ is the crust porosity and *Da* is the Damköhler number characterizing the competition between the precipitation–dissolution reactions and ion transport within the crust^26^; *ε* is the immobile bead layer porosity (taken equal to 0.36) when the travelling crust is partially embedded. After detachment, *ε* = 1*.* The evolution of the travelling crust top and bottom surface positions within the cell was computed from these equations, using the evaporation flux determined from the mass loss experimental data, and the absorption flux from the liquid level variations in the cell and the adjacent cylinder. The corresponding curves are the blue and red dashed lines labelled “Model” in Fig. 2. The agreement between the model and the experimental data represents an additional element in favor of the dissolution–precipitation mechanism. Note that *Da* has been used as a fitting parameter, equal to 2.7 in the present study. *Da* requires information such as the crust interfacial area per unit volume or the crust effective diffusion coefficient^26^ to be determined directly, i.e. not through a fitting procedure. This information is currently unavailable in literature.

Whereas the displacements of the crust top and bottom surfaces are similar after detachment, it can be seen from Fig. 2 that the bottom surface displacement is faster than the top surface displacement before the detachment. This was expected because the available space for the displacement is less when the crust bottom surface is moving in the immobile layer (this corresponds to the factor $\varepsilon$ in the relationship $\frac{d{z}_{tcb}}{dt}=\boldsymbol{ }\frac{{C}_{sat}{j}_{b}}{{\rho }_{cr}\varepsilon \left(1-{\varepsilon }_{c}\right)\left(1-{C}_{sat}\right)}\left[\frac{Da}{1+Da}\right]$ ). However, the consideration of this effect is not sufficient to explain the faster displacement of the crust bottom surface before detachment. The main factor lies in the absorption flux *j*_*t*_ which is greater in this period than the evaporation flux as discussed before and shown in the inset in Fig. 3a.

### Reduced evaporation, Kelvin effect, pore sizes on crust top surface

**Figure 7:**
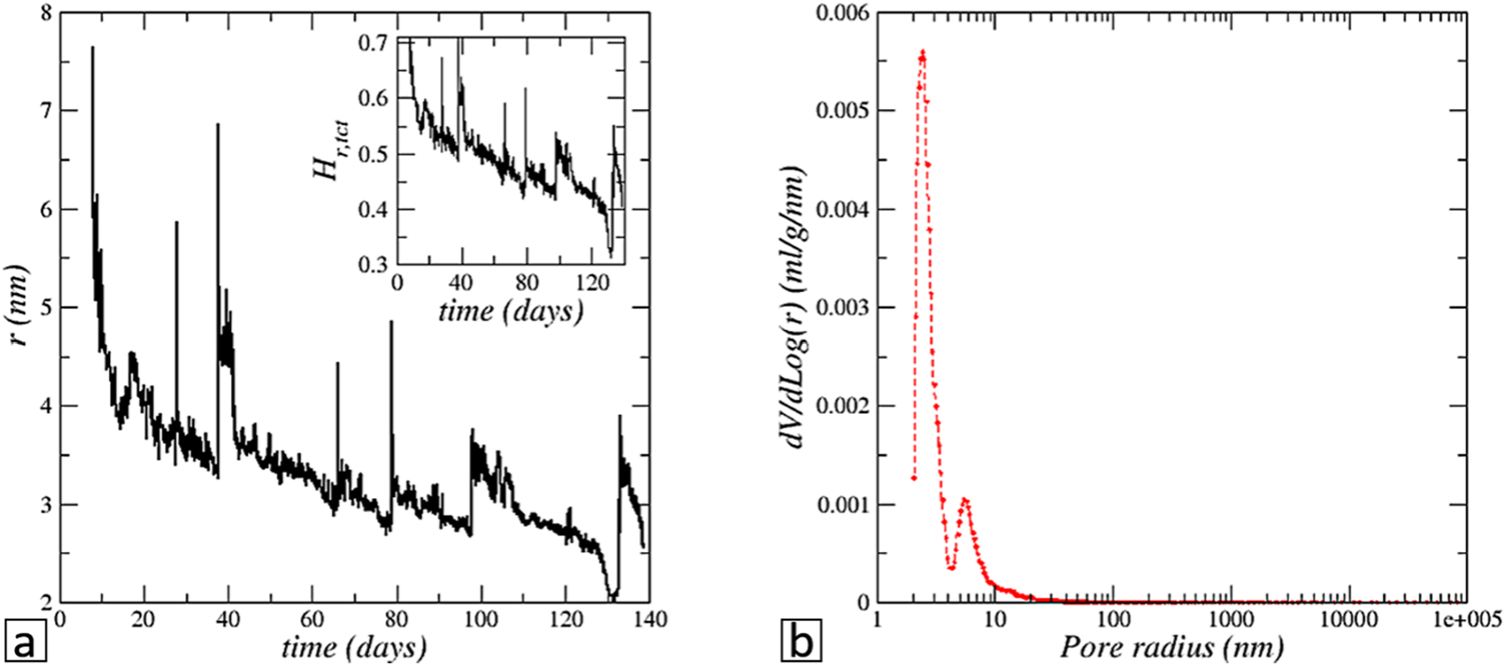
(**a**) Variation of menisci curvature radius at the crust top surface compatible with the evaporation rates observed in the experiment according to Kelvin relationship. The inset shows the computed relative humidity at the travelling crust top surface. (**b**) Salt crust pore size distribution from mercury intrusion porosimetry (see “Methods”).

Since the crust is wet and the evaporation rate is less than the potential evaporation (Fig. 3b), the conclusion is therefore that the relative humidity at the crust top surface, ${H}_{r,tct}$ , is less than ${H}_{r,sat}$ . Using again Fick’s law, the relative humidity at the crust top surface can be determined from the equation ${\frac{d{m}_{weighting}}{dt}=-A D}_{v}\frac{{M}_{v}}{RT}{p}_{vsat}\frac{\left({H}_{r,tct}-{H}_{r,inf}\right)}{{h}_{t}}$ , where both ${m}_{weighting}$ and ${h}_{t}$ are experimental data. For the long period after detachment, this leads to the results displayed in the inset in Fig. 7a. As can be seen, the average relative humidity at the travelling crust top surface is significantly lower than the relative humidity (0.75) at the interface with a saturated brine. This happens for instance during drying (see ref.^27^) when the porous medium surface is partially dry. However, the salt crust displacement is remarkably stable, the top surface topography does not change significantly while it moves upward. Therefore, the travelling crust top surface pores must be uniformly wet and not partially dry. Then the question is how the evaporation rate from a porous surface with wet pores can be significantly lower than the potential evaporation. According to the literature, two main mechanisms can explain such a vapor pressure reduction. A first option is when the surface pores are sufficiently spaced from each other. This option can be explored using a relationship proposed by Schlünder^28^, expressed as $\frac{j}{{j}_{potential}}\approx \frac{1}{1+\frac{{d}^{2}}{2\pi {r}_{p}{h}_{t}}}$ , where *d* is the mean distance between the pores and ${r}_{p}$ is the mean surface pore diameter. Using this formula leads to the desired evaporation rates only for extremely low surface porosity in the range [10^−7^–10^−6^]. Such low values are unlikely, given again that the crust top surface morphology does not change significantly during its displacement. The other mechanism which is much more likely to happen is the Kelvin effect^29^. The Kelvin effect refers to the modification of the equilibrium vapour pressure *p*_*vequ*._ due to the liquid–gas interface curvature. It can be computed from the relationship ${H}_{r,eq}=\frac{{p}_{vequ.}}{{p}_{vs}}=\mathrm{exp}\left(-\frac{{M}_{v}}{RT}\frac{2\gamma cos\theta }{{\rho }_{\mathcal{l}}r}\right)$ , where *p*_*vs*_ is the saturation vapour pressure corresponding to a flat liquid–vapour interface (thus *p*_*vs*_ = 0.75 *p*_*vsat*_ for a saturated NaCl aqueous solution), *γ* is the surface tension, *θ* the contact angle (θ ~ 0^30^), $\rho_{\ell }$ the liquid density; *r* is the liquid–gas interface curvature radius considering a pore entrance spherical shape. For the values of ${H}_{r,tct}$ reported in Fig. 7a, Kelvin relationship leads to the meniscus curvature radii depicted in Fig. 7a. As can be seen, the curvature radii are in the range (3–4 nm).

In other words, the travelling crust low evaporation rate results from the reduced partial vapour pressure at the travelling crust top surface due to the combined effect of the presence of the salt in the solution and the sufficiently high menisci curvature for the Kelvin effect to have an impact. The menisci curvature would adapt during the crust displacement so as to obtain an evaporation rate equal to the maximum possible evaporation rate, the latter corresponding to the absorption rate at the crust bottom surface.

### Mercury intrusion porosimetry

The existence of very small pores compatible with the required magnitude of the Kelvin effect, is confirmed by mercury intrusion porosimetry. In order to get pore size information from mercury intrusion porosimetry (MIP), porous salt crusts were formed using the same approach as for the Hele–Shaw cell experiment but with the glass bead packing set in a cylinder (3.6 cm in a diameter, 5 cm in height) instead of the Hele–Shaw cell. The set-up helps to obtain a representative salt crust sample at the end of the experiment. For all the samples tested, the formation of a porous crust with reduced evaporation was observed. Thus, the crusts obtained from the cylinder experiments are expected to be reasonably representative of the detached crust in the Hele–Shaw cell. Figure 6b shows the pore sizes obtained by MIP for the temperature and relative humidity the closest to the ones in the Hele–Shaw cell experiment, i.e. ~ 22 °C, ~ 40%. As can be seen, the pore sizes obtained by MIP are in quite good agreement with the theoretical estimates reported in Fig. 6a. Also the MIP indicates a crust porosity of 25%. In fact, MIP was performed on three different crust samples, all corresponding to reduced evaporation situations. MIP indicated pore sizes comparable to the ones shown in Fig. 6b for the three samples tested. Although such small pore sizes are close to the measuring limit of the machine^31^, the fact remains that the three samples examined show the same trend with the presence of pore sizes in the range of a few nanometers in diameter. Hence, the consistency of the MIP results with the pore size estimate based on the evaporation rate and the Kelvin relationship gives confidence in the explanation of the reduced evaporation.

### Crust permeability estimate

**Figure 8:**
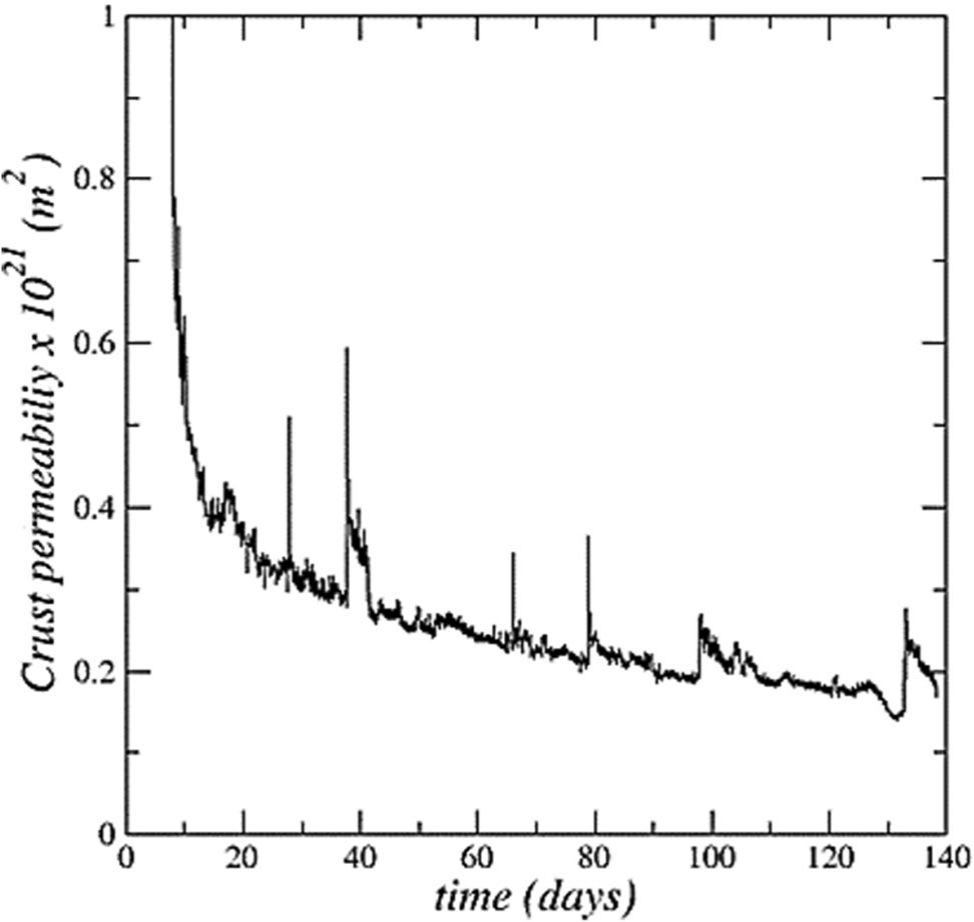
Detached crust permeability estimate as a function of time.

An estimate of the crust permeability can then be determined from the requirement that the viscous pressure drop across the crust be compatible with the meniscus curvature depicted in Fig. 7a, i.e. the capillary pressure difference between the crust top and bottom surfaces. Consider that the crust is wet and saturated by brine. Then the corresponding viscous pressure drop across the crust required to reach the desired crust top surface meniscus curvature levels is $\delta p=\frac{2\gamma cos\theta }{r}$ , assuming that the meniscus curvature radius is much larger at the crust bottom surface (since liquid water forms there one can assume that the bottom surface capillary pressure jump is negligible compared to the top surface capillary pressure jump). As shown in ref.^26^, the velocity induced in the crust can be estimated as a function of the evaporation flux as $\rho V=\left(1+\mathrm{\alpha }\right){j}_{t}$ where $\mathrm{\alpha }=\frac{{C}_{sat}\left(\rho {\varepsilon }_{c}+{\rho }_{cr}\left(1-{\varepsilon }_{c}\right)\right)}{{\rho }_{cr}\left(1-{\varepsilon }_{c}\right)\left(1-{C}_{sat}\right)}\left[\frac{Da}{1+Da}\right]$ (thus $\mathrm{\alpha }$ = 0.3 for *Da* = 2.7 and ${\varepsilon }_{c}$ = 0.25). Applying Darcy’s law, one obtains an estimate of the crust permeability *k* as, $k=\frac{{\mu (1+\mathrm{\alpha })j}_{t} {h}_{tc} r}{{\rho }2\gamma cos\theta }$ , where ${h}_{tc}$ is the travelling crust thickness (Fig. 2) and *µ* is the solution dynamic viscosity. For *θ* = 0^30^, the values of *r* depicted in Fig. 7a and the evaporation flux values in the experiment, the estimate of *k* shown in Fig. 8 is obtained. As can be seen, *k* is in the range (0.2 $\times$ 10^–21^–0.3 $\times$ 10^–21^) m^2^ over most of the detached crust displacement period. This is a quite low permeability but consistent with pore sizes below 10 nm (Fig. 7).

The crust permeability estimate is on the same order of magnitude as the values reported for salt rock permeability^32^. By contrast, the permeability reported in ref.^33^ is several orders of magnitude greater (~ 10^–12^ m^2^). The latter was measured for a salt crust forming on a coarse porous material (a sand with a mean grain diameter of 240 μm). It has been shown^13^ that the porous substrate mean pore size has a great impact on the evaporation rate in the presence of a salt efflorescence. The evaporation rate was comparable to the potential evaporation for a sufficiently coarse porous substrate whereas a significant evaporation rate reduction was obtained for a porous substrate with small mean pore size, i.e. in the micronic range in the experiments reported in ref.^13^. This is an indication that the efflorescence pore sizes are correlated to the substrate pore sizes. It was inferred that the higher the pore size in the substrate, the higher is the pore size in the efflorescence. In other words, the crust permeability is highly dependent on its formation conditions and in particular on the mean pore size of the porous medium at the surface on which it forms. From the present study and ref.^33^, it can be inferred that the salt crust permeability can vary by several orders of magnitude depending on its formation condition.

### Salt solubility in nanopores and in water in tension state

In the analysis, the possible pore size impact on salt solubility was not considered. Molecular dynamics simulations^34^ indicate that confinement in nanopores has a noticeable impact on solubility for pores less than 1 nm, thus smaller than the ones considered in this study. However, it has been reported that the solubility increases when water is in tension state due to capillary effects^35^. The capillary pressure jump for a pore of radius 3 nm (Fig. 7a), is 55 MPa. This would correspond to a 18% solubility increase^35^. The solubility increase can reduce the vapor pressure at the menisci since the greater the ion concentration the lower is the equilibrium water vapor pressure. This effect could in part explain the reduced evaporation rate in conjunction with Kelvin effect. However, this would not change the main conclusion that the crust is wet and the pore size is on the order of a few nanometers at the crust top surface.

## Discussion and conclusion

We reported on the dynamics of a salt crust with full detachment from the porous substrate. From optical visualization, mass variation measurement and theoretical considerations, we were able to explain the air gap formation, whose size is similar to field observations^18^, by dissolution–reprecipitation process driven by a vapor partial pressure gradient. In order to simplify the determination of the mass transfer rate underneath the crust from the liquid level variations in the cell, the porous medium was initially removed except for a thin upper layer. Nevertheless, the considered situation has equivalents in nature, for instance when the water table in the soil is sufficiently far away from a salt crust. However, the full detachment observed in our experiment is favorized by the Hele–Shaw confined geometry and the presence of the cell lateral walls. Nevertheless, we believe that the Hele–Shaw situation reported and analyzed in the present article is representative of the salt crust local detachments observed in nature^18^. We have also shown that the evaporation from a wet crust can be significantly lower than the potential evaporation. This result is important since it explains the very low salt crust evaporation rate often observed experimentally, without hypothesizing a dry crust that acts as a water vapor diffusion barrier^1,36,37^. Although a dry crust situation is possible, considering the crust as wet should be a better assumption in many situations. For instance, when the liquid saturation is high in the porous medium while the crust forms, a quite common situation since the liquid must be connected to the porous medium surface for the crust to form. Furthermore, the present study shows that the salt crust does not need to be hydraulically connected to the liquid phase in the porous medium to be wet. Both results, i.e. the detailed identification of an air gap formation mechanism and the reduced evaporation from a wet crust due to the formation of sufficiently small pores are new. They constitute important advances in the understanding of salt crust dynamics and associated phenomena. They open new perspectives for the analysis, understanding and control of important related phenomena such as the salinization of soils, the evaporation in the critical zone or the damages due to salt crystallization in porous materials.

## Methods

### Experiment repeatability

The paper is based on a 138-day experiment, which was in fact preceded by a 130 day long drying step during which the salt crust formed in the Hele–Shaw cell. The initial crust growth period of 130 days is long in this type of experiment due to the reduced evaporation rate. In order to have a representative volume of crust to suspend, long periods of time are required. Therefore, with the drying step, the total duration of the experiment is 268 days. Because of its quite long duration, the experiment has not been repeated under exactly the same conditions. However, as indicated below in the section on the scanned electron microscopy shorter experiments have been performed under similar conditions and have led to the same major results (salt crust upward migration and reduced evaporation). As indicated above in the section on the mercury intrusion porosimetry, the experiment has also been performed in cylindrical vessels. Again, crust upward migration and reduced evaporation were obtained. These features are therefore generic. For the article, we have chosen to consider our longest and best documented experiment.

### Image processing

Image processing was performed with Fiji^38^ using well-established filtering and general binary operations applied either globally or locally to the images. First, all the pictures were registered in order to correct for the lateral drift, which occurs when the camera runs for a long period of time. The plugin used in order to correct for this issue can be found in ref.^39^ and gives an average displacement of 0.4072 pixels (this represents the mathematical average offset applied over the whole stack). Some issues were encountered while detecting the crust bottom surface when it’s still embedded in the porous medium. To overcome this problem, a small amount of manual linking was applied to restore the interface continuity. Accuracy of the processing procedure was verified both by eye inspection and by curve plotting comparison (between registered and non-registered data sets) in order to check for inconsistences.

### Liquid level and crust limiting surface positions from processed images

Positions of crust top and bottom surfaces were measured by image processing considering 13 vertical lines of pixels evenly distributed over the cell width (the pixel lines are indicated by small white arrows in Fig. 1b). Due to some local salt creeping over the cell wall toward the end of the experiments (the salt creeping is visible in Fig. 1b in the rightmost picture); the three rightmost pixel lines were eventually discarded. Therefore, positions shown in the figures and considered in the analysis correspond to arithmetic averages over ten vertical pixel lines. The liquid level in the adjacent cylinder and cell were determined using an identical procedure considering one vertical pixel line (by the cell wall and for the cylinder at the meniscus middle).

### Scanned electron microscopy (SEM)

SEM images in Fig. 1 have been obtained when both layers were deemed dry. Note that the samples used to obtain the images are from a similar experiment with a shorter overall duration: 27 days for forming the suspended crust and 21 days of crust displacement. The high vacuum SEM (HIROX Tabletop SEM SH-4000M) analysis has been done after proper treatment of the samples (drying), in the same conditions as during the experiment, this giving a necessary technical time between the end of the experiment and analysis of about 4 months.

### Mercury intrusion porosimetry (MIP)

MIP was performed with a Micrometics AutoPore IV 9500 apparatus. The device measures the volume of mercury invading the porous sample while the mercury pressure is increased step by step. For a given pressure step, the pore radius is estimated from Young–Laplace equation assuming cylindrical pores. Defining the specific invaded volume *V* as the mercury intrusion volume divided by the volume of the sample, the pore size distribution is obtained by plotting the differential volume $\frac{dV}{dLog(r)}$ as a function of the pore size obtained from the Young–Laplace equation as exemplified in Fig. 7b. More information on the technique can be found in ref.^31^.

## Supplementary Information

Supplementary Information.

Supplementary Video 1.

## Data Availability

Data are available from the corresponding author on reasonable request.

## References

[1] Fujimaki H, Shimano T, Inoue M, Nakane K (2006). Effect of a salt crust on evaporation from a bare saline soil. Vadose Zone J..

[2] Gran M, Carrera J, Olivella S, Saaltink MW (2011). Modeling evaporation processes in a saline soil from saturation to oven dry conditions. Hydrol. Earth Syst. Sci..

[3] Nachshon U, Weisbrod N, Dragila MI, Grader A (2011). Combined evaporation and salt precipitation in homogeneous and heterogeneous porous media. Water Resour. Res..

[4] Bergstad M, Or D, Withers PJ, Shokri N (2017). The influence of NaCl concentration on salt precipitation in heterogeneous porous media. Water Resour. Res..

[5] Fox GA, Wilson GV (2010). The role of subsurface flow in hillslope and stream bank erosion: A review. Soil Sci. Soc. Am. J..

[6] Nachshon U (2016). Seepage weathering impacts on erosivity of arid stream banks: A new conceptual model. Geomorphology.

[7] Rengasamy P (2006). World salinization with emphasis on Australia. J. Exp. Bot..

[8] Jamil A, Riaz S, Ashraf M, Foolad MR (2011). Gene expression profiling of plants under salt stress. Crit. Rev. Plant Sci..

[9] Lubelli B, Van Hees RPJ, Groot CJWP (2004). The role of sea salts in the occurrence of different damage mechanisms and decay patterns on brick masonry. Constr. Build. Mater..

[10] Flatt RJ, Caruso F, Aguilar Sanchez AM, Scherer GW (2014). Chemomechanics of salt damage in stone. Nat. Commun..

[11] Schiro M, Ruiz-Agudo E, Rodriguez-Navarro C (2012). Phys. Rev. Lett..

[12] Sghaier N, Prat M (2009). Effect of efflorescence formation on drying kinetics of porous media. Transp. Porous Media.

[13] Eloukabi H, Sghaier N, Ben Nasrallah S, Prat M (2013). Experimental study of the effect of sodium chloride on drying of porous media: The crusty-patchy efflorescence transition. Int. J. Heat Mass Trans..

[14] Norouzi Rad M, Shokri N, Sahimi M (2013). Pore-scale dynamics of salt precipitation in drying porous media. Phys. Rev. E.

[15] Gupta S, Huinink HP, Prat M, Pel L, Kopinga K (2014). Paradoxical drying due to salt crystallization. Chem. Eng. Sci..

[16] Veran-Tissoires S, Prat M (2014). Evaporation of a sodium chloride solution from a saturated porous medium with efflorescence formation. J. Fluid Mech..

[17] Shokri-Kuehni SMS, Vetter T, Webb C, Shokri N (2017). New insights into saline water evaporation from porous media: Complex interaction between evaporation rates, precipitation, and surface temperature. Geophys. Res. Lett..

[18] Nachshon U, Weisbrod N, Katzir R, Nasser A (2018). NaCl crust architecture and its impact on evaporation: Three-dimensional insights. Geophys. Res. Lett..

[19] Siedel H (2018). Salt efflorescence as indicator for sources of damaging salts on historic buildings and monuments: A statistical approach. Environ. Earth Sci..

[20] Grementieri L, Molari L, Derluyn H, Desarnaud J, Cnudde V, Shahidzadeh N, de Miranda S (2017). Numerical simulation of salt transport and crystallization in drying Prague sandstone using an experimentally consistent multiphase model. Build. Environ..

[21] Dai S, Shin H, Santamarina JC (2016). Formation and development of salt crusts on soil surfaces. Acta Geotech..

[22] Van Enckevort WJP, Los JH (2013). On the creeping of saturated salt solutions. Cryst. Growth Des..

[23] Qazi MJ, Salim H, Doorman CAW, Jambon-Puillet E, Shahidzadeh N (2020). Salt creeping as a self-amplifying crystallization process. Sci. Adv..

[24] Hobeika N, Bouriat P, Touil A, Broseta D, Brown R, Dubessy J (2017). Help from a hindrance: Using astigmatism in round capillaries to study contact angles and wetting layers. Langmuir.

[25] Owens JS (1926). Condensation of water from the air upon hygrosopic crystals. Proc. R. Soc. A.

[26] Licsandru G, Noiriel C, Duru P, Geoffroy S, Abou-Chakra A, Prat M (2019). Dissolution-precipitation-driven upward migration of a salt crust. Phys. Rev. E.

[27] Attari Moghaddam A, Kharaghani A, Tsotsas E, Prat M (2018). A pore network study of evaporation from the surface of a drying non-hygroscopic porous medium. AIChE J..

[28] Schlünder EU (1988). On the mechanism of the constant drying rate period and its relevance to diffusion controlled catalytic gas phase reactions. Chem. Eng. Sci..

[29] Adamson AW (1990). Physical Chemistry of Surfaces.

[30] Abell AB, Willis KL, Lange DA (1999). Mercury intrusion porosimetry and image analysis of cement-based materials. J. Colloid Interface Sci..

[31] Corti T, Krieger UK (2007). Improved inverted bubble method for measuring small contact angles at crystal-solution-vapor interfaces. Appl. Opt..

[32] Cosenza Ph, Ghoreychi M, Bazargan-Sabet B, de Marsily G (1999). In situ rock salt permeability measurement for long term safety assessment of storage. Int. J. Rock Mech. Min. Sci..

[33] Pietrowski J, Huisman JA, Nachshon U, Pohlmeier A, Vereecken H (2020). Gas permeability of salt crusts formed by evaporation from porous media. Geosciences.

[34] Malani A, Ayappa KG, Murad S (2006). Effect of confinement on the hydration and solubility of NaCl in water. Chem. Phys. Lett..

[35] Hulin C, Mercury L (2019). Capillarity-driven supersolubility in dual-porosity systems. Geochim. Cosmochim. Acta.

[36] Desarnaud J, Derluyn H, Molari L, de Miranda S, Cnudde V, Shahidzadeh N (2015). Drying of salt contaminated porous media: Effect of primary and secondary nucleation. J. Appl. Phys..

[37] Eloukabi H, Sghaier N, Prat M, BenNassrallah S (2011). Drying experiments in a hydrophobic model porous medium in the presence of a dissolved salt. Chem. Eng. Technol..

[38] Schindelin J, Arganda-Carreras I, Frise E (2012). Fiji: An open-source platform for biological-image analysis. Nat. Methods.

[39] Preibisch S, Saalfeld S, Schindelin J, Tomancak P (2010). Software for bead-based registration of selective plane illumination microscopy data. Nat. Methods.

