# Supplementary material for: Detachment mechanism and reduced evaporation of an evaporative NaCl salt crust
Source: Sci Rep. 2022 May 6;12:7473. doi: 10.1038/s41598-022-11541-w (PMC9076668; doi:10.1038/s41598-022-11541-w)
Supplement: Supplementary file 1 — Supplementary Information. [file 41598_2022_11541_MOESM1_ESM.docx]

**Supplementary information**

S1. Movie: Upward migration of salt crust due to dissolution-precipitation leading to full detachment
